# Senescence as a manifestation of Mirror Autoprosopometamorphopsia

**DOI:** 10.1192/j.eurpsy.2023.1442

**Published:** 2023-07-19

**Authors:** S. Kalita, D. Birwatkar, A. Hirsch

**Affiliations:** 1 Psychiatry; 2 Spartan Health Sciences University, Vieux Fort, Saint Lucia; 3 Psychiatry, Smell and Taste Treatment & Research Institue, Chicago, United States

## Abstract

**Introduction:**

Obligate autoscopic mirror hallucinations of senescence have not heretofore been described.

**Objectives:**

To reveal that perception of looking older in the mirror may be the manifestation of Mirror Autoprosopometamorphopsia.

**Methods:**

A 37 year old right handed female, with schizoaffective disorder, bipolar subtype She described that when she would gaze at herself in the mirror, she would not see her current face, but rather the visage of an “old person”. This would recur whenever she would directly look at herself in the mirror, and would avoid glancing at any mirrors because she was fearful of looking at her transform senescent countenance. She realised it was not another person but rather herself in the future, having become her geriartric self.

**Results:**

Abnormalities in Physical Examination: Mental Status Examination: Hyperverbal, grandiose with expansive affect, poor insight and judgment. Recalls 3 out of 4 objects in 3 minutes and all 4 with reinforcement. Proverb testing: correct abstraction. Neuropsychiatric Testing: The Patient Health Questionnaire 9:7 (mild depression). Other: Magnetic Resonance Imaging/ Magnetic Resonance Angiography of Brain with Infusion: Normal.

**Conclusions:**

Autoscopic mirror hallucinations appearing only when embedded in a mirror are obligate autoscopic mirror hallucinations and suggest occipital and parietal lobe dysfunction (Virk, 2018). The inability to recognize the perception of another image or another person replacing the individual looking in the mirror, while defined as a mirror sign, may also be viewed as “a capgras syndrome for the mirror image” (Feinberg, 2005). Distortion of one’s own face only when viewed in a mirror is autoprosopometamorphopsia. With such distortion, this may be a misidentification of one’s own image. This phenomenon is classified as a form of delusional misidentification syndrome with inability to recognise one’s image in the mirror (Postal, 2005). Autoprosopometamorphopsia, obligate to mirror reflection, but metamorphosized to enhance perceived senescence, has not been specifically localized. Possibly a single lesion in the non dominant inferior parietal lobe may have caused this phenomenon. Somatoparaphrenia with somatosensory illusions involving body image are seen with parietal lobe dysfunction (Nightingale, 1982). In the general population, an individual’s focus on a mild facial imperfection often is associated with a negative view of their image. Exaggeration of this to involve the entire face, with projection of imperfection of aging, may be a somatic manifestation of such negative self image. It is possible that such senescent autoprospometamorphopsia may be prevalent, to a lesser degree, in the general population and may be a nidus for younger people seeking cosmetic and plastic surgical intervention of the face.

**Disclosure of Interest:**

None Declared

